# (-)-Epicatechin protects against myocardial ischemia/reperfusion injury via autophagy-dependent ferroptosis

**DOI:** 10.18632/aging.205477

**Published:** 2024-01-25

**Authors:** Kong Junhong, Tsai Yun, Shui Guangxing, Ding Yuhan, Xiang Qian, Zhang Haowen

**Affiliations:** 1 Nanjing University of Chinese Medicine Affiliated Changzhou Hospital, Changzhou 213000, Jiangsu, China; 2 Nanjing University of Chinese Medicine, Nanjing 210023, Jiangsu, China; 3 Department of Nephrology, Shanghai Jiading Hospital of Traditional Chinese Medicine, Shanghai 201899, China

**Keywords:** (-)-Epicatechin, myocardial ischemia reperfusion, ROS, autophagy, ferroptosis

## Abstract

Aim: (-)-Epicatechin (EPI) has physiological activities such as antioxidant, anti-inflammatory and immune enhancement. In this study, we elucidated the protective effects of EPI in myocardial ischemia/reperfusion injury (MI/RI) and its mechanisms.

Methods: An *in vivo* I/R model was constructed by performing left anterior descending coronary artery surgery on rats, and an *in vitro* I/R model was constructed by subjecting hypoxia/reperfusion treatment on H9C2 cells. The damage of cardiac tissues was detected by 2,3,5-triphenyltetrazolium chloride (TTC) and hematoxylin-eosin (H&E) staining, and expressions of ferroptosis-related proteins were examined by Western blot. Changes in the number of autophagosomes, the levels of oxidative stress and Fe^2+^ were also examined.

Results: EPI reduced abnormal electrocardiogram waveform and infarct size caused by MI/RI in rats. The increasing trend of levels of reactive oxygen species (ROS) and Fe^2+^ was reversed by EPI, suggesting that EPI can reduce ferroptosis *in vivo*. Moreover, the levels of lipid ROS and LC3 in H9C2 cells were decreased with EPI treatment, and autophagy and ferroptosis were also alleviated in a dose-dependent manner *in vitro*. Co-cultivation of USP14 inhibitor IU1 and EPI further revealed that EPI regulates ferroptosis through the USP14-autophagy pathway.

Conclusions: EPI can reduce the level of oxidative stress by promoting USP14 to reduce autophagy, thus inhibiting autophagy dependent ferroptosis and reducing oxidative stress, and has a protective effect on myocardial infarction/myocardial infarction.

## INTRODUCTION

Ischemic heart disease is responsible for millions of deaths worldwide, and blood reperfusion can be an effective remedy for myocardial infarction [1]. Currently, percutaneous coronary intervention and thrombolytic therapy are used to restore blood supply to the ischemic myocardium, but rapid blood flow restoration will further damage the myocardium and contribute to severe myocardial ischemia reperfusion injury (MI/RI) [2]. Therefore, the clinical concern about MI/RI has attracted widespread attention. Recent studies have reported the existence of myocardial cell injury induced by autophagy in MI/RI, in addition, ferroptosis is also a mechanism of myocardial cell injury induced by this model [3–5]. Excessive autophagy can promote ferroptosis by degrading ferritin and increasing the concentration of free iron ions in cells [6].

Iron is an essential element in living cells [7]. Ferroptosis is characterized by the down-regulation of phospholipid hydroperoxidase GPX4, the accumulation of iron and the production of lipid peroxidation [8, 9]. Abnormal accumulation of Fe^2+^ causes excessive free radical production through the Fenton reaction, which may lead to specific cell death [8]. Fe^3+^ is stored in ferritin, which consists of 24 subunits that regulate iron content and immune regulation [10]. Iron can be recruited from ferritin using ferritinophagy in the case of iron deficiency or increased iron demand, and nuclear receptor coactivator 4 (NCOA4) has been identified as a crucial regulator in ferritinophagy. Autophagy degrades and recycles cytoplasmic proteins and organelles and play an essential role in maintaining intracellular homeostasis. Microtubule-associated protein light chain 3 (LC3) protein, a membrane marker for autophagosomes, is widely used to detect autophagosomes formation by observing the intensity of green fluorescent protein (GFP) puncta in cells transduced with the LC3 gene fused to GFP [11]. Studies have shown that excessive autophagy caused by cardiac I/R injure cascade can cause tissue damage [12, 13].

(-)-Epicatechin (EPI) is a naturally occurring plant flavanol compound with the chemical formula C_15_H_14_O_6_ that is widely found in tea, cocoa and herbs [14]. It has an important antioxidant function, which is the binding of phenolic hydroxyl groups in the molecule and free radicals to achieve free radical scavenging effects. Study has shown that moderate consumption of chocolate can reduce the risk of cardiovascular disease, and these effects seem to be mediated by EPI [15]. In addition, several studies have demonstrated flavonoid-induced cardioprotective effects in animal models or myocardial I/R cell lines [16, 17]. However, the protective effects of EPI on MI/RI have not been clearly elucidated.

In the present study, a rat myocardial ischemia-reperfusion model treated with ligation and reperfusion of the left anterior descending coronary artery (LAD) surgery was established *in vivo*, and a hypoxic repletion treated H9C2 cell model was established *in vitro* to assess the protective effect of EPI on MI/RI. By analyzing the differences in the expressions of key proteins related to autophagy and ferroptosis, as well as changes in oxidative stress levels, the protective effect of EPI on MI/RI were revealed, and the mechanism by which EPI alleviates MI/RI was clarified, intending to explore a potential drug for the treatment of MI/RI.

## MATERIALS AND METHODS

### Reagents and animals

(-) Epicatechin (SE8100, HPLC ≥ 98%) was purchased from Solarbio Technology Co. (Beijing, China). Adult male Sprague-Dawley rats (SCXK20190010, SPF (Beijing) Biotechnology Co., Ltd., China) weighing 250-300 g were used after one week of temporary housing. Rats were randomly divided into five groups (n=12 in each group): control group, rats receive normal feeding without LAD surgery; I/R group, rats were pretreated with saline by oral gavage for 15 days prior to ligation and reperfusion of the LAD surgery; I/R + L-EPI group, rats were pretreated with 1 mg/kg/day of EPI by oral gavage for 15 days prior to ligation and reperfusion of the LAD surgery [18]; I/R + H-EPI group, rats were pretreated with 2 mg/kg/day of EPI by oral gavage for 15 days prior to ligation and reperfusion of the LAD surgery; DIL group, rats were pretreated with 20 mg/kg/day diltiazem by oral gavage for 15 days prior to ligation and reperfusion of the LAD surgery.

Prior to surgery, animals were anesthetized with sodium pentobarbital (60 mg/kg, *i.p.*). The electrocardiogram (ECG) needle electrodes were subcutaneously implanted into the limbs of rats to observe and record standard body region lead ECG. The LAD procedure was then performed with ligation and reperfusion, occluding the LAD allowing myocardial ischemia for 45 min before reperfusion. Mice were executed by intraperitoneal injection of an overdose of sodium pentobarbital, and for biochemical analysis, the blood was collected from the abdominal aorta and stored in the refrigerator at -80° C for subsequent analysis. The heart was removed for later use.

### Pathomorphological detection of cardiac tissue

The heart tissue was cut into 5 slices, 2,3,5-Triphenyltetrazolium chloride (TTC, Sigma, USA) staining was used to stain slices evenly for 30 min at 37° C. This staining could distinguish the viable myocardium (red) and the nonviable myocardium (white). Photos were taken to quantify the infarction rate by Image J. The calculation method of infarct rate is: $\text{infarct}\;\text{rate}=\frac{\text{infarct}\;\text{area}}{\text{total}\;\text{area}}\times 100\%$.

Paraffin sections were stained using hematoxylin and eosin, and then the cardiac tissues sections were dehydrated. The sections were sealed and placed under a microscope (Olympus, Tokyo, Japan) for observation and evaluation of cardiac tissue lesions.

### Determination of malondialdehyde (MDA), superoxide dismutase (SOD), iron content and reactive oxygen species (ROS) in cardiac tissues

The content of Malondialdehyde (MDA) in heart tissue was measured using Malondialdehyde content detection kit (BC0025, Solarbio) and the absorbance of the supernatant of the heart tissue samples was measured at 600 nm according to the manufacturer’s instructions. The absorbance of supernatant of heart tissue were detected by Superoxide dismutase (SOD) activity detection kit (BC0175, Solarbio) at 425 nm and the SOD activity of the samples was calculated. The total iron levels were measured in heart tissue in each group using the Iron Assay kit (ab83366, Abcam, UK). Tissues homogenate was lysed in 4 volume of buffer and centrifuged at 16,000 × g for 10 min. 5 μL of iron reducer agent was added into 50 μL of samples for Fe^2+^ assay, and 100 μL of iron probe solution was added to the samples and incubated at 25° C for 60 min kept in dark place. The absorbance of samples at 593 nm was measured using a micro spectrophotometer (Nanodrop, Thermo Fisher Scientific, USA).

ROS of cells were detected by Dihydroethidium (DHE) reactive oxygen ROS fluorescent probe (S0063, Beyotime, China) following the manufacturers’ instructions.

### Western blotting

Cardiac tissues and cardiomyocytes extracts were used for Western blot analysis. The equal amount of protein samples obtained from tissues or cells were separated by SDS-PAGE electrophoresis and then transferred to PDVF membrane. The membranes were incubated with the following primary antibodies: USP14 (1191S, CST, USA), Beclin1 (3738, CST), LC3 (12741, CST), NCOA4 (ab86707, Abcam) and FTH1 (3998, CST) and transferrin receptor 1 (TfR1; ab1086, Abcam). Secondary antibodies were incubated and then visualized with the ECL Plus assay Kit (K002, Affinity, USA).

### Cell culture and model

H9C2 cells were purchased from Sigma (USA) and cultured in Dulbecco’s modified Eagle’s medium (DMEM, Gibco, USA) supplemented with 100 μg/mL of penicillin and 100 μg/mL of streptomycin (Beyotime, China) and 10% fetal bovine serum (FBS, Wisent Inc., Montreal, Canada) at 37° C with 5% CO_2_.

H9C2 cells were incubated with 100 uL EPI at different concentrations (1, 2.5, 5, 10, 20, 40, 100 μM) for 24 h, and MTT assays were performed to obtain the appropriate concentration of EPI.

The H9C2 cells at logarithmic growth stage were spread in 96-well plates and cultured in 5% CO_2_ and 37° C carbon dioxide incubator. After 24 h, H9C2 cells were washed and low-sugar serum-free DMEM culture was added. The culture was placed in an incubator under anoxic conditions (5% CO_2_, 1% O_2_, 94% N_2_) for 24 h at 37° C. The cells were then taken out and replaced with DMEM medium containing 10% FBS in an incubator at 37° C under re-oxygenation conditions (5% CO_2_, 21% O_2_, 74% N_2_) for 2, 4, and 8h to obtain the appropriate re-oxygenation time [19]. When the survival rate was 50-60%, it meant that the oxygen-glucose deprivation/regain (OGD/R) modeling was successful.

Cells were divided into five groups: control group, OGD/R group (OGD/R model), 2.5-EPI (OGD/R + 2.5 μM EPI), 5-EPI (OGD/R + 5 μM EPI), and 10-EPI (OGD/R + 10 μM EPI). EPI-treated groups were treated with 2.5, 5, and 10 μM EPI for 24 h and then incubated under hypoxia for 24 h and re-oxygenation for 2h, respectively. Cell viability was detected by MTT.

### Determination of iron content, ROS, MDA and SOD in myocardial cells

H9C2 was inoculated in 60 mm petri dishes (1.5-2×10^6^ cells). Cells in each group were collected after EPI treatment at corresponding concentrations, and operated as section 2.3 mentioned above.

### Morphological observation and Western blot of cardiomyocytes

H9C2 cells were cultured in 60 mm petri dishes (1.5-2×10^6^ cells). After corresponding treatment according to Section 2.3, cells were collected for observing autophagosome formation of cardiomyocytes and detecting expressions of autophagy related proteins in cardiomyocytes by Western blotting.

### Detection of lysosomes and autophagosomes

The microtubule associated protein light chain 3 (LC3) protein is a membrane marker of autophagosome. Therefore, autophagosomes formation was detected by detecting the GFP fluorescence intensity of cells transduced with the fused GFP-LC3 gene. H9C2 cells were seeded at a concentration of 4 ×10^4^ cells/well in 24-well plate, and then Ad-GFP-LC3b (C3006, Beyotime, China) adenovirus transfection was performed followed by adherent culture for 24 h. After transfection for 24 h, the corresponding cultures were replaced.

Lysosomes were detected using the Lyso-Tracker Red fluorescent probe (C1046, Beyotime), an eosinophilic fluorescent probe for the labelling and tracing of acidic organelles within living cells. According to the manufacturer’s instruction, the cell culture medium was removed, the prepared Lyso-Tracker Red staining working solution was added and co-incubated with the cells for 60 min at 37° C. The Lyso-Tracker Red staining working solution was removed and fresh cell culture medium was added, followed by observation with a fluorescent microscope.

### Ubiquitylation assay

Co-IP assays were performed as previously described. Protein A/G Plus Sepharose beads were added to the tissue or cell lysate according to the manufacturer’s instructions and the immunoprecipitates were collected. The immunoprecipitates were boiled and centrifuged to precipitate the agarose beads. The supernatant was used for Western blot of the target protein using a Beclin 1 K63-linked specific polyubiquitin (12930, CST) antibody.

### Statistical analysis

All data are expressed as the means ± standard deviation (S.D.). T-test was used to compare the data between the two groups, ANOVA was used to compare data among three groups. Statistics were performed using GraphPad Prism 8.02. Unless otherwise stated, results were considered to be statistically significant for values *P<0.05, **P<0.01 compared to the I/R or OGD/R group.

### Availability of data and materials

All data generated or analysed during this study are included in this published article.

## RESULTS

### EPI alleviates myocardial ischemia-reperfusion injury *in vivo*

**Figure 1:**
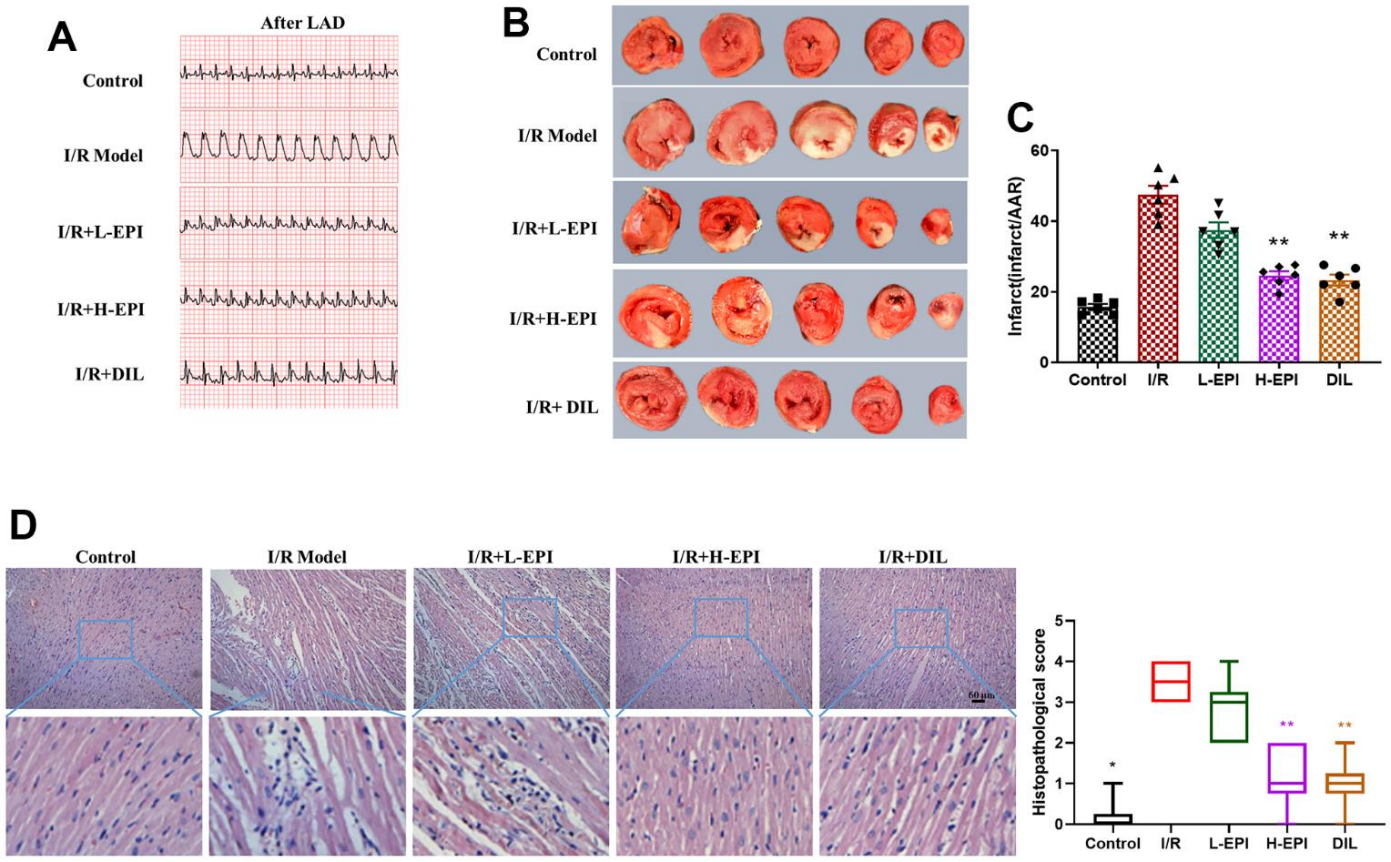
**EPI alleviates myocardial ischemia-reperfusion injury *in vivo*.** (**A**) Electrocardiogram of each group of rats after modeling. (**B**) TTC staining of cardiac tissues in each group. (**C**) Infarction rates of cardiac tissues in each group. (**D**) Effects of EPI on histopathological changes of cardiac tissues suffered from surgical procedures of ischemia-reperfusion, and histogram scores of myocardial tissue of rats in each group. I/R + L-EPI: 1 mg/kg/day of EPI; I/R + H-EPI: 2 mg/kg/day of EPI. Data (n=6) are expressed as mean ± SD, *P < 0.05 compared with I/R group, **P < 0.01 compared with I/R group.

Abnormal fluctuations in the ECG are considered to be arrhythmia. Figure 1A showed the two-dimensional images respectively. Increased wave amplitudes were observed in the model group compared with the control group. These features were typical symptoms of ventricular arrhythmias, indicating that I/R induced obvious cardiomyocytes injury. The wave amplitude was significantly decreased by treatment with EPI. The results of TTC staining were shown in Figure 1B, the white area in the I/R group was obviously larger than that in the control group (*P<0.05*), indicating that the *in vivo* MI/RI model was established successfully. Notably, the white areas in the EPI and DIL groups were significantly smaller than that in the I/R group (Figure 1B, 1C). In Figure 1D, the control group had normal myocardial fiber arrangement and no inflammatory cells, while the I/R model group exhibited disorganized myocardial structure with significant inflammatory cell infiltration. Rats treated with EPI showed generally regular fiber structure and less inflammatory cell infiltration compared to the I/R group. Histogram scores also suggested that EPI significantly improved inflammatory cell infiltration in myocardial tissue. In addition, EPI treatment also significantly decreased LVDEP, CK-MB, and LDH and increased +dp/dt, LVSP, and -dp/dt, suggesting that EPI improved ventricular systolic and diastolic function (Supplementary Figure 1).

### EPI increases antioxidant properties in cardiac tissues

**Figure 2:**
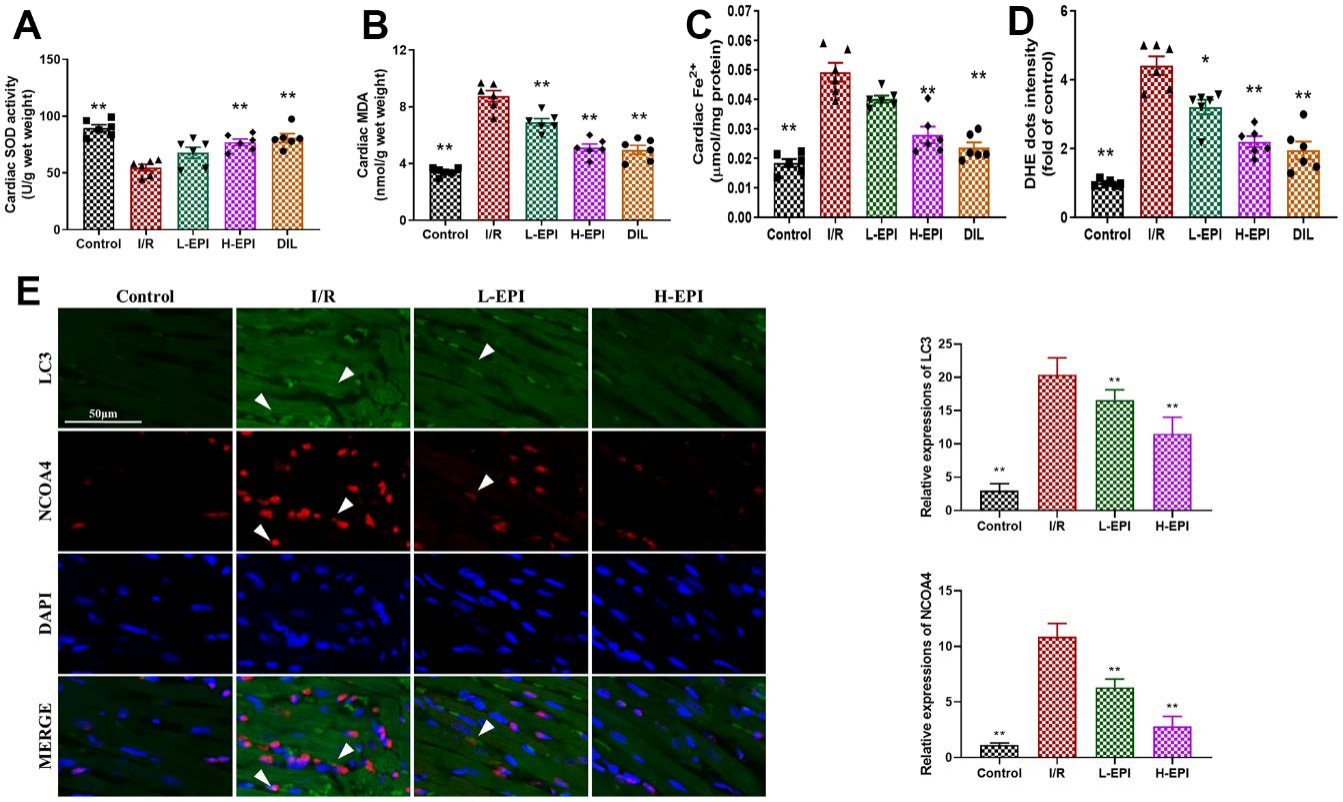
**EPI increases antioxidant properties in cardiac tissues.** (**A**) The activity of SOD in cardiac tissues. (**B**) The content of MDA in cardiac tissues. (**C**) The concentration of Fe^2+^ in cardiac tissues. (**D**) The content of ROS in cardiac tissues. (**E**) The fluorescent intensity of LC3 and NCOA4 in myocardial tissues. I/R + L-EPI: 1 mg/kg/day of EPI; I/R + H-EPI: 2 mg/kg/day of EPI. Data (n=6) are expressed as mean ± SD, *P < 0.05 compared with I/R group, **P < 0.01 compared with I/R group.

The level of oxidative stress is considered to be an important indicator for studying reperfusion-induced injury. Thus, the contents of SOD, MDA, ROS and Fe^2+^ were detected in cardiac tissues. Compared with the control group, the SOD activity of the I/R group were decreased significantly (*P<0.05*), while the MDA, ROS and Fe^2+^ values of the I/R group were increased respectively (Figure 2B–2D). After the treatment of EPI (1 or 2 mg/kg) and DIL, their MDA, ROS and Fe^2+^ contents decreased significantly, while SOD values increased significantly (Figure 2A), and high concentration of EPI were more effective in alleviating oxidative stress. The results showed that EPI played a protective role by significantly reducing the content of MDA and restoring the content of antioxidant enzyme SOD, indicating its ability to reduce ROS-dependent tissue damage. In addition, we examined the co-expression of LC3 and NCOA4 to characterise ferritinophagy. As shown in Figure 2E, NCOA4 and LC3 were highly expressed in the I/R group, which was reduced by EPI.

### EPI inhibits the flux of autophagy and ferroptosis related proteins in cardiac tissues

**Figure 3:**
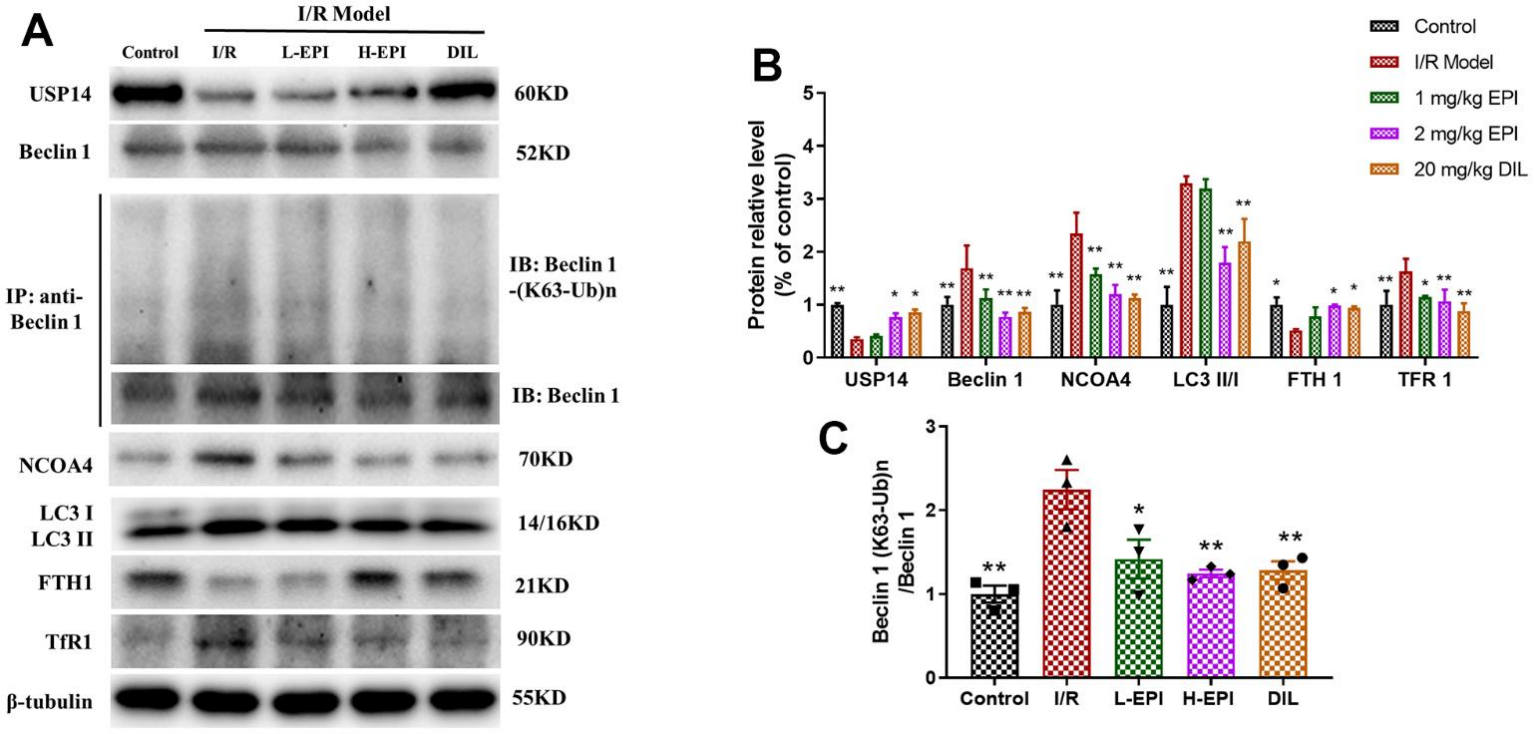
**EPI regulates the expression of autophagy and ferroptosis related proteins in cardiac tissues.** (**A**) Western blot analysis of USP14, Beclin 1, NCOA4, LC3 I, LC3 II, FTH1 and TfR1, Co-IP analysis of ubiquitination of K63 on Beclin 1. (**B**) Densitometric analysis of the bands was presented as the relative ratio of USP14, Beclin 1, NCOA4, LC3 I, LC3 II, FTH1, TfR1. (**C**) Densitometric analysis of the bands was presented as the relative ratio of K63 ubiquitination on Beclin 1. I/R + L-EPI: 1 mg/kg/day of EPI; I/R + H-EPI: 2 mg/kg/day of EPI. Data (n=3) are expressed as mean ± SD, *P < 0.05 compared with I/R group, **P < 0.01 compared with I/R group.

Autophagy and ferroptosis were regulated by ROS and oxidative stress, we detected the expressions of autophagy and ferroptosis related proteins to explore their functions in EPI protection. I/R suppressed the expressions of USP14, FTH1, and increased the expressions of Beclin1, LC3II/I and TfR1, which were significantly reversed by EPI treatment, suggesting that EPI inhibited autophagy and ferroptosis in MI/RI rat model (Figure 3A, 3B). The changes in the ubiquitination of upstream Beclin1 at K63 confirmed that MI/RI activated the Beclin 1 pathway to induce autophagy, which was downregulated by EPI treatment (Figure 3A, 3C).

### EPI increases the viability and antioxidant properties of H9C2 cells

**Figure 4:**
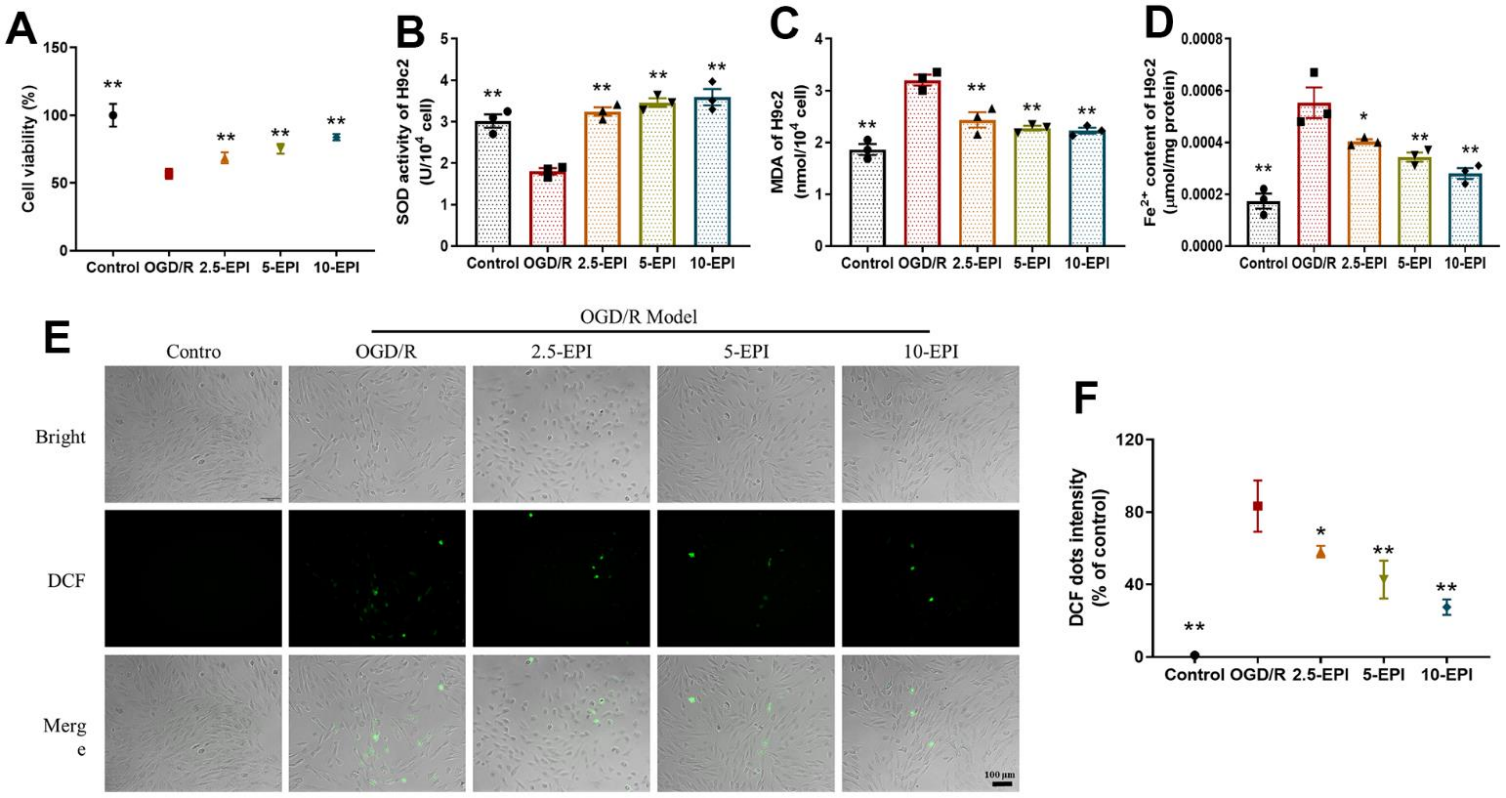
**EPI increases the viability and antioxidant properties of H9C2 cells.** (**A**) Cell viability of H9C2 cells. (**B**) The activity of SOD in H9C2 cells. (**C**) The content of MDA in H9c2 cells. (**D**) The concentration of Fe^2+^ in H9C2 cells. (**E**) The fluorescent intensity of ROS (probe-DCF) in H9C2 cells. (**F**) Histogram of ROS content of H9C2 cells. 2.5-EPI: 2.5 μM EPI; 5-EPI: 5 μM; 10-EPI: 10 μM EPI. Data (n=3) are expressed as mean ± SD, *P < 0.05 compared with OGD/R group, **P < 0.01 compared with OGD/R group.

In order to further clarify the protective mechanism of EPI on cardiomyocytes, we established an OGD/R model in H9C2 cells. In Figure 4A, EPI significantly improves cell viability with the OGD/R treatment in a concentration-dependent manner (*p<0.05*). The content of antioxidant enzyme SOD (Figure 4B) was decreased and the contents of lipid peroxidation product MDA (Figure 4C) and ROS (probe-DCF, Figure 4E, 4F) were increased in OGD/R model cells. After treatment with EPI, this trend was reversed. The content of Fe^2+^ in OGD/R group was higher than that in the control group, and significantly lower than EPI treated group (*p<0.05*, Figure 4D). These results revealed that treatment of EPI could decrease the accumulation of Fe^2+^ and increase antioxidant properties of OGD/R H9C2 cells.

### EPI down-regulates the expression of GFP-LC3 in H9C2 cells and lysosomal

**Figure 5:**
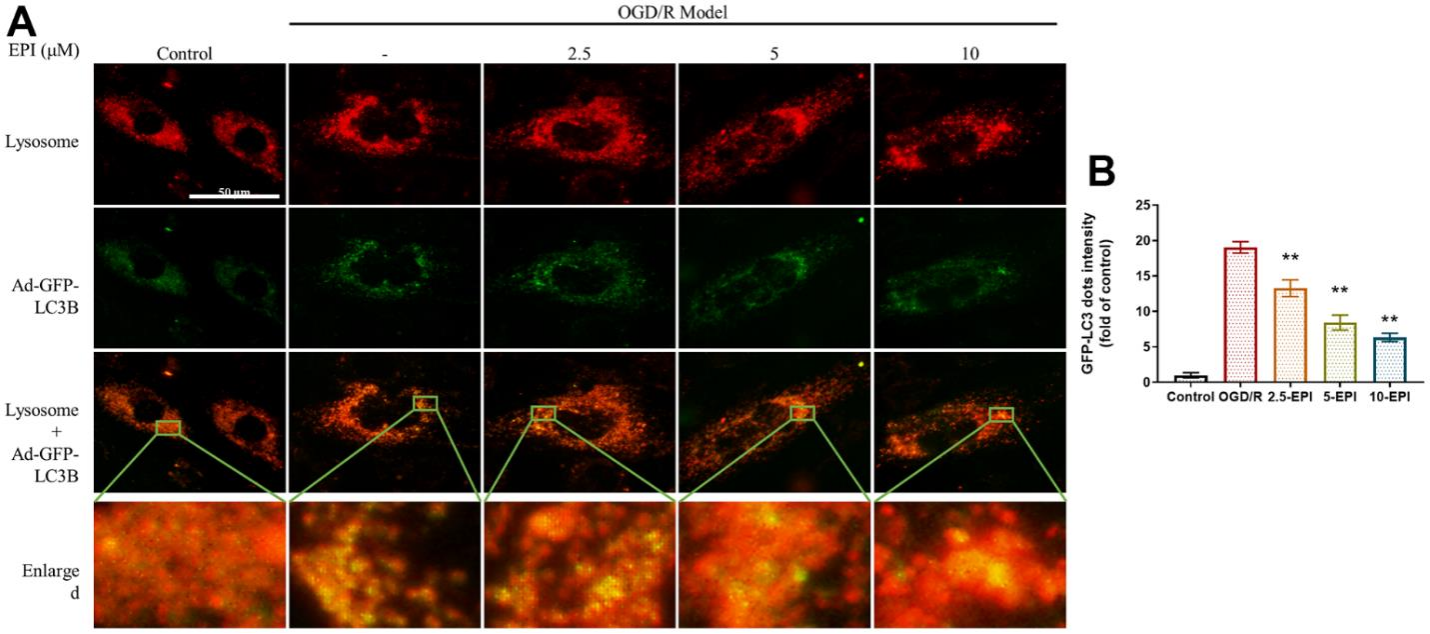
**EPI up-regulates the expression of GFP-LC3 in H9C2 cells and lysosome.** (**A**) Representative fluorescent images of GFP-LC3 and lysosomal co-staining in H9C2 cells. (**B**) GFP-LC3 dots intensity in H9C2 cells. 2.5-EPI: 2.5 μM EPI; 5-EPI: 5 μM; 10-EPI: 10 μM EPI. Data (n=3) are expressed as mean ± SD, *P < 0.05 compared with OGD/R group, **P < 0.01 compared with OGD/R group.

Measurement of autophagy were imaged using an adenoviral vector encoding GFP-LC3. After formation, autophagic vesicles fuse with lysosomes for degradation. As shown in Figure 5A, stronger yellow fluorescence indicated more binding of lysosomes and autophagosomes and more intense autophagy. EPI treatment attenuated OGD/R-induced autophagy, as the OGD/R group had significantly fewer green fluorescent puncta than the EPI-treated group (Figure 5B).

### EPI inhibits the flux of autophagy and ferroptosis related proteins in H9C2 cells

**Figure 6:**
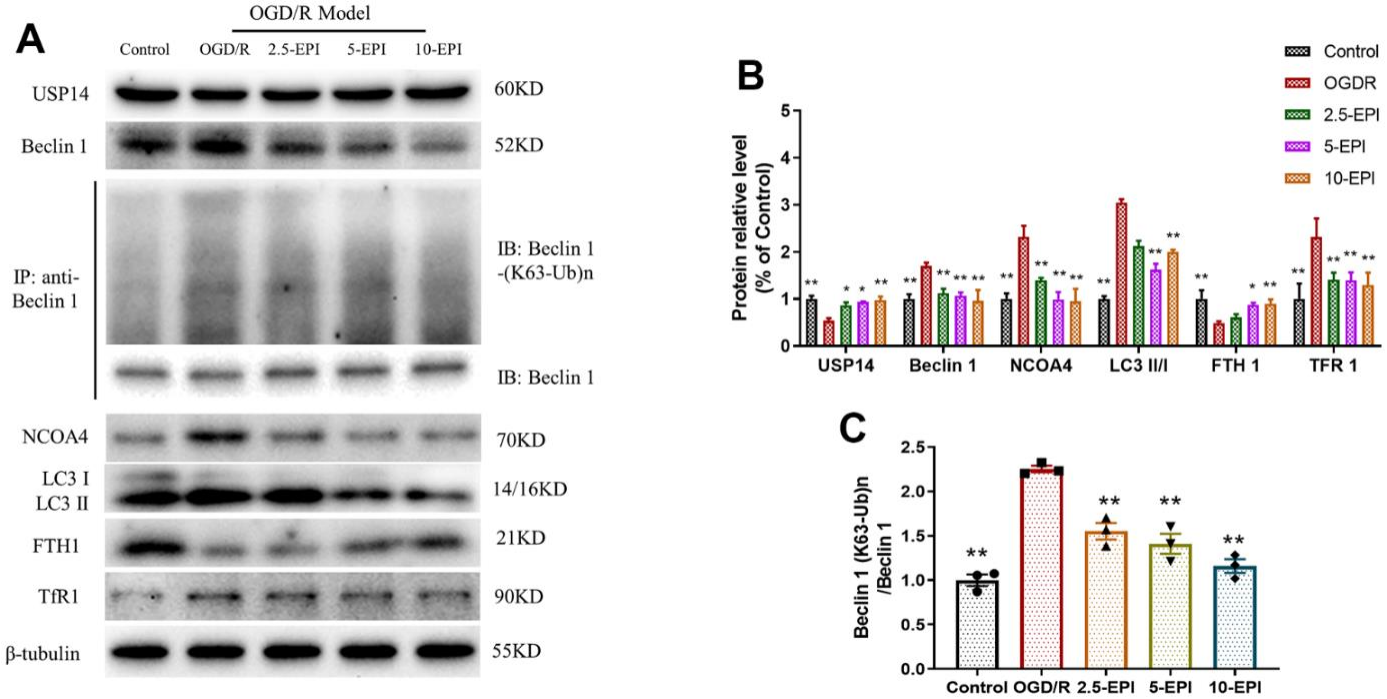
**EPI regulates the expression of autophagy and ferroptosis related proteins in H9C2 cells.** (**A**) Western blot analysis of USP14, Beclin 1, NCOA4, LC3 I, LC3 II, FTH1 and TfR1, Co-IP analysis of ubiquitination of K63 on Beclin 1. (**B**) Densitometric analysis of the bands was presented as the relative ratio of USP14, Beclin 1, NCOA4, LC3 I, LC3 II, FTH1, TfR1. (**C**) Densitometric analysis of the bands was presented as the relative ratio of K63 ubiquitination on Beclin 1. 2.5-EPI: 2.5 μM EPI; 5-EPI: 5 μM; 10-EPI: 10 μM EPI. Data (n=3) are expressed as mean ± SD, *P < 0.05 compared with OGD/R group, **P < 0.01 compared with OGD/R group.

OGD/R treatment suppressed the expressions of USP14, NCOA4 and FTH1, and increased the expressions of Beclin1, LC3II/I and TfR1, which was significantly reversed by EPI treatment, suggesting EPI inhibits autophagy and ferroptosis in *in vitro* cell model (Figure 6A, 6B). The changes in the ubiquitination of upstream Beclin1 at K63 confirmed that I/R activated the Beclin 1 pathway to induce autophagy, which was downregulated by EPI treatment (Figure 6B, 6C).

### EPI inhibits autophagy via suppressing USP14 in H9C2 cells

**Figure 7:**
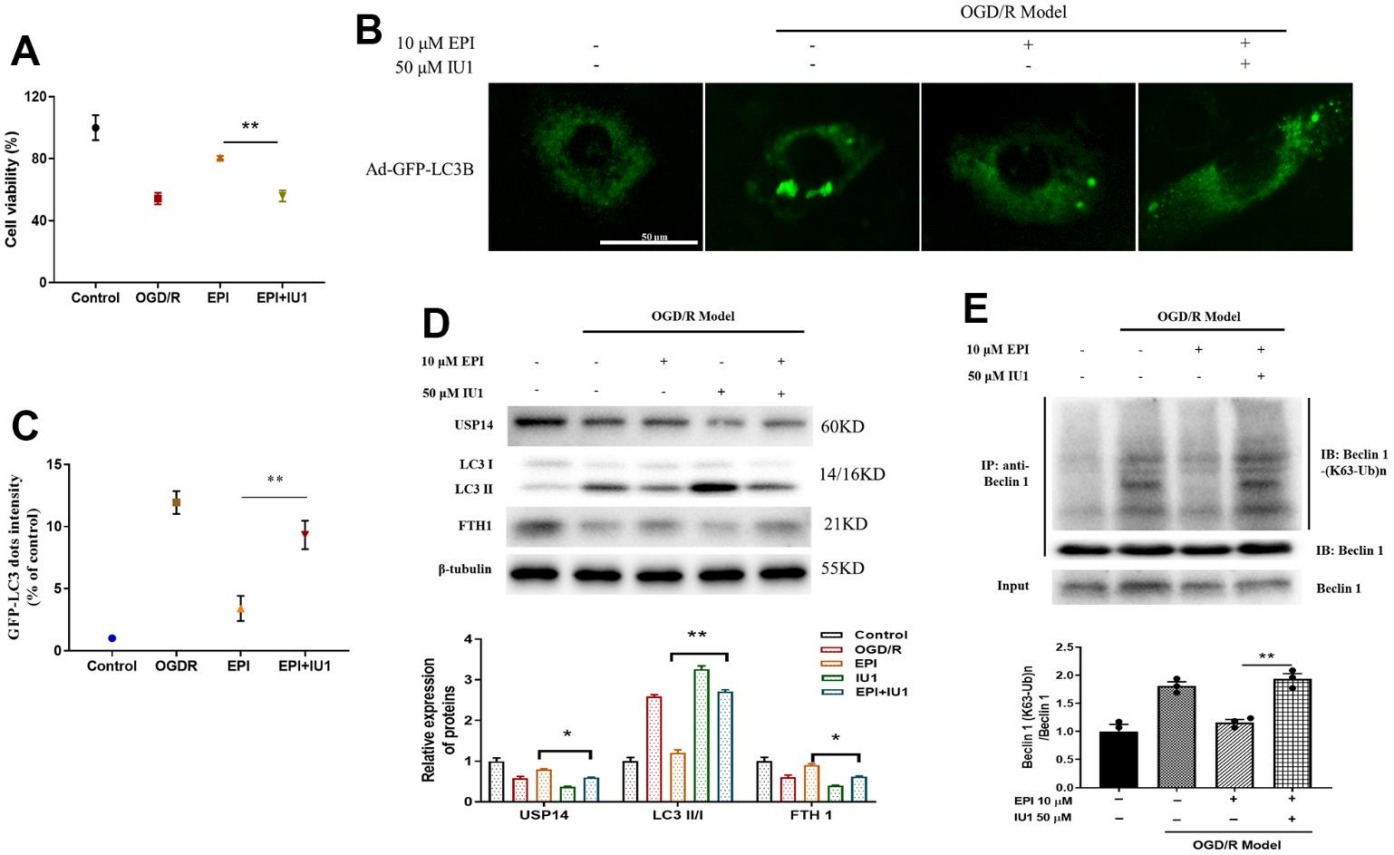
**EPI inhibits autophagy in H9C2 cells.** (**A**) Cell viability of H9C2 cells. (**B**) Representative images of GFP-LC3 green fluorescence and lysosomal red fluorescence superimposed staining in H9C2 cells. (**C**) Relative quantitative positivity of GFP-LC3. (**D**) Western blot analysis of USP14, LC3 I, LC3 II and FTH1. (**E**) Co-IP analysis of ubiquitination of K63 on Beclin 1. Data (n=3) are expressed as mean ± SD, *P < 0.05, **P < 0.01.

To study whether EPI could protect cells by regulating the expression of USP14, we used the USP14 inhibitor IU1 to explore this further. In Figure 7A, the cell viability of the EPI group was higher than that of the OGD/R group. The viability of the EPI + IU1 group was significantly reduced compared with the EPI group (*P<0.05*). Previous experiments revealed that EPI treatment could alleviate OGD/R-induced autophagy, but cell viability was obviously reduced when the inhibitor IU1 was added to co-culture (*P<0.05*, Figure 7B, 7C). EPI suppressed the expressions of LC3II/I and TfR1, and increased the expression of USP14, which was significantly reversed by EPI + IU1 treatment, suggesting EPI inhibits autophagy via suppressing USP14 expression (Figure 7D). In Figure 7E, results indicated that EPI protected cells by inhibiting the ubiquitination of K63 on Beclin1. The above results indicated that EPI promoted the expression of USP14 by promoting Beclin1 K63 ubiquitination, thereby inhibiting autophagy.

## DISCUSSION

MI/RI refers to myocardial injury caused by ischemia in myocardial tissue after there is blood perfusion again. MI/RI may lead to irreversible injury to cardiomyocytes and reduce the viability of cardiomyocytes [20]. Therefore, it is important to discover drugs that can alleviate MR/RI and their mechanisms. EPI can prevent cardiovascular disease and improve blood vessel health. The aim of this study was to elucidate the targets and mechanisms of EPI on MI/RI. This study showed that treatment with EPI could reduce lipid peroxidation and iron accumulation at the site of MI/RI. Treatment with USP14 inhibitors (IU1) revealed that EPI could target and promote the expression of USP14, activate its downstream signaling pathways to inhibit autophagy, and suppressed oxidative stress, iron accumulation and ferroptosis, and realize protective treatment of MI/RI.

MI/RI injury can cause changes in cardiac parameters, which in turn cause changes in cardiac function [21]. One of the common complications of ischemic heart disease is impairment induced by abnormal heart function. Flavonoids can exert protective effects on myocardial tissue during MI/RI, either by reducing oxidative stress, anti-inflammatory or myocardial function modulation [22]. In the present study, the results of *in vivo* experiment in MI/RI model also consistently showed that EPI pretreatment prior to I/R injury can improve cardiac function. In the clinical management of ischemic heart disease, MDA is an important marker to reflect the extent of tissue injury caused by oxidative stress [23]. At the same time, the cells themselves produce the antioxidant enzyme SOD to prevent themselves from potential damage caused by ROS [24]. As MI/RI is mainly caused by excessive accumulation of ROS, drugs targeting ROS will be effective to reduce oxidative stress at the injury lesion [25]. In the *in vivo* experiments of this study, the level of SOD was decreased and the level of MDA was increased after MI/RI modeling, implying an increase of oxidative stress. In contrast, EPI treatment reversed the decrease in SOD and the increase in MDA, significantly reduce the tissue damage by inhibiting the level of oxidative stress.

Ferroptosis is a new form of cell death dependent on iron and triggered by lipid peroxidation [4]. In recent years, a growing body of evidence has established the important role of ferroptosis in the pathogenesis of cardiomyopathy. Ferroptosis-like cell death found in heart failure rats and isoproterenol-induced H9C2 cells [26]. Ferroptosis has been reported to be involved in MI/RI-induced cardiac injury and iron chelation therapy has been reported to protect myocardium from injury significantly [27].

It has been suggested that NCOA4 plays a key role in ferritin phagocytosis, which promotes degradation of autophagic ferritin and the release of Fe^2+^ [28]. Autophagy has an important role in the death of cardiomyocytes. On the one hand, autophagy is a myocardial protective mechanism under stress conditions such as ischemia and energy deprivation; and on the other hand, excessive autophagy leads to cardiomyocyte death. In the present study, to further analyze the mechanism of EPI in the ferroptosis of cardiomyocytes, the expression of autophagy and ferroptosis related proteins were measured by Western blot. Results showed that EPI treatment could decrease the levels of NCOA4 and FTH1 *in vivo* and *in vitro*. Fluorescence results showed that OGD/R enhanced autophagy and iron content in cells, while EPI inhibited this phenomenon. The results further indicated that ferroptosis is autophagy dependent. The microtubule associated protein LC3 was an autophagosomal membrane marker and participated in the formation of autophagosomes. In this study, we found that ferroptosis was activated in H9C2 cells by OGD/R injury, and immunofluorescence results showed that OGD/R enhanced cellular autophagy and iron content, while EPI inhibited this.

Notably, EPI treatment promoted the expression of USP14, suggesting that the blocking effect of EPI on autophagy may act by promoting USP14, which was consistent with previous study [29]. The combined use of IU1 and EPI together to culture OGD/R-modelled cells revealed that the levels of LC3 and FTH1, which were originally reduced by EPI, were instead increased. Autophagy is a key regulator of ferritin degradation and ferroptosis [30]. The results further suggest that ferroptosis in MI/RI is autophagy-dependent.

There are some limitations in this study which need to be noted. Firstly, autophagy pathways may differ between species, although they have been validated from cellular experiments to animal models, and subsequent applicability in clinical studies requires attention. Secondly, the autophagy-dependent iron death we identified is only one of the EPI-mediated pathways, and it cannot be ruled out that there are other pathways that are affected thereby ameliorating myocardial ischemia/reperfusion injury. The above limiting points need to be considered together to ensure the scientific reliability of the findings and the potential for clinical application.

## CONCLUSIONS

Our study demonstrated that treatment with EPI could effectively protect ischemia-reperfused myocardial tissue and cells by increasing USP14 to reduce Beclin1-dependent autophagy, thereby down-regulating the autophagy dependent ferroptosis.

## Supplementary Material

Supplementary Figure 1
