# Supplementary material for: (-)-Epicatechin protects against myocardial ischemia/reperfusion injury via autophagy-dependent ferroptosis
Source: Aging (Albany NY). 2024 Jan 25;16(3):2181–93. doi: 10.18632/aging.205477 (PMC10911377; doi:10.18632/aging.205477)
Supplement: Supplementary Figure 1 [file aging-16-205477-s001.pdf]

SUPPLEMENTARY FIGURE

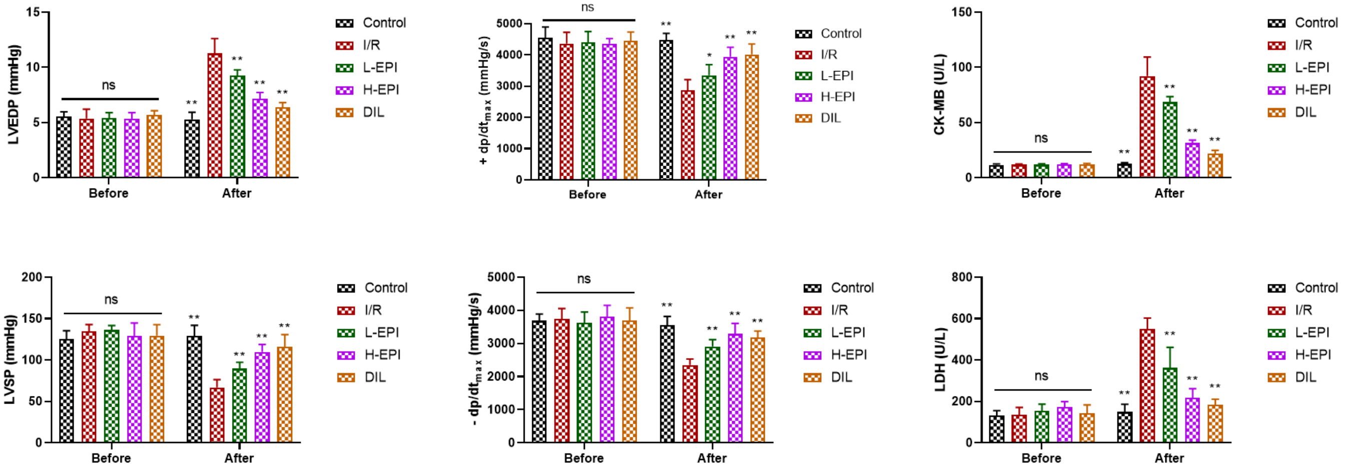

Supplementary Figure 1. The value of LVDEP, +dp/dt, CK-MG, LVSP, -dp/dt and LDH of serum in each group before and after reperfusion.
